# The impact of *CREBRF* rs373863828 Pacific-variant on infant body composition

**DOI:** 10.1038/s41598-024-59417-5

**Published:** 2024-04-17

**Authors:** Francesca Amitrano, Mohanraj Krishnan, Rinki Murphy, Karaponi A. M. Okesene-Gafa, Maria Ji, John M. D. Thompson, Rennae S. Taylor, Tony R. Merriman, Elaine Rush, Megan McCowan, Lesley M. E. McCowan, Christopher J. D. McKinlay

**Affiliations:** 1 Liggins Institute, University of Auckland, Auckland, New Zealand; 2 Department of Obstetrics and Gynaecology, University of Auckland, Auckland, New Zealand; 3 Department of Medicine, University of Auckland, Auckland, New Zealand; 4 Department of Epidemiology, University of North Carolina at Chapel Hill, Chapel Hill, NC USA; 5 Te Whatu Ora, Counties Manukau, Auckland, New Zealand; 6 Maurice Wilkins Centre for Molecular Biodiscovery, University of Auckland, Auckland, New Zealand; 7 Department of Paediatrics: Child and Youth Health, University of Auckland, Auckland, New Zealand; 8 Department of Microbiology and Immunology, University of Otago, Dunedin, New Zealand; 9 Division of Clinical Immunology and Rheumatology, University of Alabama at Birmingham, Birmingham, AL USA; 10 Faculty of Health and Environmental Science, Auckland University of Technology, Auckland, New Zealand

**Keywords:** Intrauterine growth, Obesity, Genetics research, Paediatric research, Diabetes, Gestational diabetes, Type 2 diabetes

## Abstract

In Māori and Pacific adults, the *CREBRF* rs373863828 minor (A) allele is associated with increased body mass index (BMI) but reduced incidence of type-2 and gestational diabetes mellitus. In this prospective cohort study of Māori and Pacific infants, nested within a nutritional intervention trial for pregnant women with obesity and without pregestational diabetes, we investigated whether the rs373863828 A allele is associated with differences in growth and body composition from birth to 12–18 months’ corrected age. Infants with and without the variant allele were compared using generalised linear models adjusted for potential confounding by gestation length, sex, ethnicity and parity, and in a secondary analysis, additionally adjusted for gestational diabetes. Carriage of the rs373863828 A allele was not associated with altered growth and body composition from birth to 6 months. At 12–18 months, infants with the rs373863828 A allele had lower whole-body fat mass [FM 1.4 (0.7) vs. 1.7 (0.7) kg, aMD −0.4, 95% CI −0.7, 0.0, P = 0.05; FM index 2.2 (1.1) vs. 2.6 (1.0) kg/m^2^ aMD −0.6, 95% CI −1.2,0.0, P = 0.04]. However, this association was not significant after adjustment for gestational diabetes, suggesting that it may be mediated, at least in part, by the beneficial effect of *CREBRF* rs373863828 A allele on maternal glycemic status.

## Introduction

Gestational diabetes mellitus (GDM) is characterised by impaired glucose tolerance and/or impaired fasting glucose concentrations first diagnosed during pregnancy^1^. Key risk factors for GDM include maternal overweight and obesity, excessive pregnancy weight gain, low socioeconomic status, unbalanced diet and genetic background^2^. The genetic contribution to GDM is of increasing interest, given the finding in several genome-wide association studies of strongly reproducible susceptibility variants for type 2 diabetes and GDM, such as *CDKAL1* and near *MTNR1B*, suggesting that these conditions may have a shared genetic background^3–5^. This is further supported by the fact that women who develop GDM have up to a sevenfold increased risk of subsequently developing type 2 diabetes^6^.

Recently, a missense variant in the CREB3 Regulatory Factor (*CREBRF*) gene (rs373863828, Arg457Gln, c.1370G > A) was identified as being strongly associated with higher body mass index (BMI, + 1.4 kg/m^2^) and waist circumference (+ 3 cm), but an approximately two-fold reduced likelihood of type 2 diabetes among adults of Polynesian^7,8^ and Micronesian^9^ ancestry. In New Zealand, the rs373863828 minor A allele is prevalent among Māori and Pacific people with a frequency of 10% to 27% but it is rare among other ethnic groups, e.g., 0.01% in East Asians and 0.004% in Europeans^10^. In young adult New Zealand Māori and Pacific men (mean age 28 years), the rs373863828 A allele was associated with lower circulating levels of the muscle inhibitory hormone myostatin and reduced fat mass^11^. In Samoan infants and children, the rs373863828 A allele was associated with higher lean mass at 2 to 4 months^12^ and a taller stature at 5 to 18 years of age^13^. Consistent with its protective role in diabetes^8–10^, the rs373863828 A allele was associated with a five-fold reduction in the likelihood of GDM in New Zealand Māori and Pacific pregnant women with obesity, without any apparent influence on gestational weight gain or maternal lipid concentrations^14^.

It is unclear if fetal carriage of the rs373863828 A allele alters neonatal and infant growth and body composition independent of its effects on maternal glucose homeostasis. The presence of such an association could have important implications for the interpretation of metabolic studies in pregnancy in Māori and Pacific populations. Therefore, among Māori and Pacific infants exposed to maternal obesity and a high prevalence of GDM, we investigated the relationship between *CREBRF* rs373863828 genotype and body size, composition and growth from birth to 12 to 18 months of age. We hypothesised that Māori and Pacific infants carrying the rs373863828 A allele, compared to those without the allele, would have increased growth in weight and length at 12 to 18 months but a leaner body composition.

## Methods

### Study population

This study was undertaken among infants born to women in the Healthy Mums and Babies (HUMBA) Trial (ACTRN12615000400561, first registration 29/4/2015) conducted in South Auckland, New Zealand, where more than half of the maternity population is of Māori or Pacific descent^15^. Ethical approval was obtained from the Southern Health and Disability ethics committee, New Zealand (14/STH/205) and the study was performed in accordance with the Declaration of Helsinki. The HUMBA Trial had a 2 × 2 factorial design and investigated in pregnant women with obesity (BMI ≥ 30 kg/m^2^) whether excessive gestational weight gain and birthweight in their infants could be reduced by: (1) a multi-faceted dietary intervention provided by Community Health Workers compared with routine dietary advice; and/or (2) probiotics compared with placebo^15^. Women with pre-existing diabetes [HbA_1c_ ≥ 50 mmol/mol (≥ 6.7%)] in early pregnancy were excluded. The dietary intervention, which consisted of four home-based education sessions, reinforced with behaviour change techniques, personalised pregnancy weight gain targets and motivational text messaging, had a modest effect on reducing gestational weight gain but did not affect infant birthweight. Probiotics did not alter either of the co-primary outcomes.

Infants were eligible for this study if their mother or father self-identified as being of Māori or Pacific (Polynesian) descent, and their parent/guardian provided written informed consent for genetic testing. Genomic deoxyribonucleic acid (DNA) was collected from buccal samples at follow-up at either of two time points, 5 to 6 months or 12 to 18 months, using the Oragene saliva kit (DNA Genotek Inc., Ottawa, Canada), and was extracted using the PureLink Genomic DNA Mini Kit (Invitrogen, USA). DNA samples were stored at −80 °C prior to genetic testing.

A custom designed TaqMan probe-set (Applied Biosystems, USA) was created for rs373863828, as previously described^14^, using a custom Python script (snp_design; DOI: 10.5281/zenodo.56250) to annotate the human genome build 37 reference sequence (ftp://ftp.ensembl.org/pub/grch37, accessed 1 August 2016) with rs373863828 and any surrounding SNPs (obtained from the NCBI dbSNP build 147 common SNP list; ftp://ftp.ncbi.nlm.nih.gov/snp):

forward primer: CAAGAGAGGATGCTGAGACCAT;

reverse primer: ACCATGATGTAAGCCATTTTTCTGATACA;

probe 1 (VIC): TGAGTGGAACC**G**AGATAC;

probe 2 (FAM): AGTGGAACC**A**AGATAC.

Genotyping was performed using the LightCycler 480 Real-Time PCR System in 384-well plates (Roche Applied Science, USA). Quality control measures included genotyping of non-template controls (to ensure the absence of cross-contamination and primer cross-reactivity) and genotyping of samples set as technical replicates to evaluate consistency. There was 100% successful genotyping call rate. Re-genotyping of 25% of the samples demonstrated 100% concordance.

Anthropometric and body composition measurements were performed in infants at birth and 5 to 6 months and 12 to 18 months’ corrected age, as previously described^15^. Briefly, weight was measured to 10 g accuracy by electronic scale; length to 1 mm by infantometer; circumferences for head, left arm (mid-acromio-radiale), chest and abdomen to 1 mm by lasso tape; and triceps and subscapular skinfold thickness to 0.2 mm by Holtain calipers. Whole-body fat and lean mass (FM, LM) were measured at birth and 5 to 6 months by air displacement plethysmography (PEA POD, Cosmed, Concord, USA) and at 12 to 18 months by hand-to-foot bioimpedance spectroscopy analysis (SFB7, Impedimed, Queensland, Australia).

### Statistical analysis

Analysis was performed using SAS v9.4 (SAS Institute, Cary, NC, USA). Socioeconomic deprivation was defined as being in the lowest national quintile for New Zealand Deprivation Index, calculated using maternal address at study recruitment^16^. Customised birthweight centiles were calculated using the New Zealand version of GROW^17^. Sex- and corrected-age-specific z-scores for anthropometric measures at birth were calculated from the United Kingdom World Health Organization (UK-WHO) preterm population reference and subsequently from the WHO 2006 Child Growth Standard^18^. Small for gestational age (SGA) and LGA were defined as < 10th or > 90th birthweight centile, respectively, for customised and population references. Left arm muscle area was calculated from arm circumference and triceps skinfold thickness^19^. Whole-body FM and LM were converted to height indices (FMI, LMI kg/m^2^) to account for the body size. Conditional growth in weight and length from birth and 5 months to 12 to 18 months’ corrected age was assessed as conditional growth SS, calculated from the residuals of the regression of the current z-scores on previous z-scores^20^.

In the primary analysis (model 1), the carriage of the rs373863828 A allele was tested for association with body size and composition at birth, 5 to 6 months’ and 12 to 18 months’ corrected age, and growth in weight and length from birth to 12 to 18 months’ using generalised linear models, adjusted for potential confounding by gestational age, sex, ethnicity and parity. Estimated exposure effects are presented as adjusted mean difference (aMD) or adjusted odds ratio (aOR). We estimated that a study of 70 infants with an allelic ratio of AAA/AG to GG of 1:2 would have 80% power to detect a 0.7 standard deviation (SD) difference in continuous measures.

In secondary analysis (model 2), regression models were additionally adjusted for potential mediation by maternal GDM status using the diagnostic thresholds of the International Association of Diabetes in Pregnancy Study Groups (IADPSG)^21^. For this analysis, only infants whose mothers completed an oral glucose tolerance test were included, to ensure accurate diagnosis of GDM. In post hoc exploratory analysis, the primary model was further adjusted for the HUMBA trial interventions.

## Results

Of 140 HUMBA infants followed up at 12 to 18 months corrected age, 67 were of Māori or Pacific descent and had consent provided for genetic testing. Eighteen (27%) were carriers for the *CREBRF* rs373863828 A allele, with two (11%) being homozygous (Table1). Approximately half of infants with the rs373863828 A allele had a mother with the variant allele, consistent with Mendelian inheritance. Infants with and without the rs373863828 A allele were similar for maternal age, parity, body mass index and smoking; socioeconomic deprivation; sex; ethnicity; mode of birth and gestation length (Table 1). Although not statistically significant, mothers of infants carrying the rs373863828 A allele were three times less likely to have GDM (Table 1), consistent with our previous report^14^.

**Table 1:** Characteristics of infants and their mothers.

Characteristics	Total	N	Infant CREBRF rs373863828 genotype				P
			A/G or A/A*	N	G/G	N	
Maternal characteristics							
rs373863828 genotype		67		18		49	0.01
A/A	2 (3.0%)		2 (11.1%)		0 (0.0%)		
A/G	12 (17.9%)		6 (33.3%)		6 (12.2%)		
G/G	48 (71.6%)		9 (50.0%)		39 (79.6%)		
Unknown	5 (7.5%)		1 (5.6%)		4 (8.2%)		
Age, years	29.0 (5.7)	67	30.1 (5.9)	18	28.5 (5.6)	49	0.30
Nulliparous	14 (20.9%)	67	3 (16.7%)	18	11 (22.5%)	49	0.74
Smoking in pregnancy	19 (28.4%)	67	6 (33.3%)	18	13 (26.5%)	49	0.76
BMI, kg/m^2^	39.8 (6.7)	67	41.1 (5.9)	18	39.4 (7.0)	49	0.37
30.0 to 34.9	13 (19.4%)	67	3 (16.7%)	18	10 (20.4%)	49	
≥ 35.0	54 (80.6%)	67	15 (83.3%)	18	39 (79.6%)	49	
GDM (IADPSG)	15 (26.3%)	57	2 (11.8%)	17	13 (32.5%)	40	0.19
OGTT plasma glucose concentration at 24–28 weeks, mmol/L							
Fasting	4.7 (0.4)	57	4.6 (0.4)	17	4.7 (0.5)	40	0.30
1 h	8.0 (1.5)	41	8.1 (1.4)	12	8.0 (1.6)	29	0.79
2 h	6.2 (1.2)	57	6.0 (1.7)	17	6.2 (1.0)	40	0.65
Socioeconomic deprivation^#^	57 (85.1%)	67	16 (88.9%)	18	41 (83.7%)	49	0.72
HUMBA trial interventions							
Dietary intervention	35 (52.2%)	67	8 (44.4%)	18	27 (55.1%)	49	0.58
Probiotic capsule	31 (46.3%)	67	8 (44.4%)	18	23 (46.9%)	49	1.00
Infant characteristics							
Ethnicity		67		18		49	1.00
Māori	25 (37.3%)		7 (38.9%)		18 (36.7%)		
Pacific	42 (62.7%)		11 (61.1%)		31 (63.3%)		
Sex, female	31 (46.3%)	67	7 (38.9%)	18	24 (49.0%)	49	0.58
Caesarean birth	18 (26.9%)	67	7 (38.9%)	18	11 (22.4%)	49	0.22
Gestation length, weeks	39.7 (1.4)	67	39.5 (1.6)	18	39.7 (1.3)	49	0.67
< 37 weeks	2 (3.0%)	67	1 (5.6%)	18	1 (2.0%)	49	
≥ 37 weeks	65 (97.0%)	67	17 (94.4%)	18	48 (98.0%)	49	

Data are number (percent) or mean (standard deviation) ^a^Homozygous (A/A) n = 2. ^b^New Zealand Deprivation Index (2013) decile 8 to 10^19^.

*A* CREBRF rs373863828 minor allele, *BMI* body mass index, *GDM* gestational diabetes mellitus, *IADPSG* International Association of Diabetes in Pregnancy Study Groups, *OGTT* oral glucose tolerance test.

P value is for the comparison between genotype groups (A/G or A/A vs. GG) using Fisher exact test or Student t-test. To convert glucose concentration from mmol/L to mg/dL divide by 0.0555.

In primary analysis, infant carriage of the rs373863828 A allele was not significantly associated with body size at birth, including weight and length z-scores, BMI or being LGA, nor measures of body composition, including skinfold thickness, arm muscle area or whole-body FM and LM, and associated indices (Table 2). Similarly, at 5 to 6 months’ corrected age, infant carriage of the rs373863828 A allele was not significantly associated with body size or composition (Table 3). In secondary post hoc analysis, results were not appreciably altered by adjusting the primary model for exposure to the HUMBA Trial interventions.

**Table 2:** Body size and composition at birth.

Outcomes	Infant CREBRF rs373863828 genotype				Adjusted model 1 aMD or aOR (95% CI)	P	Adjusted model 2 aMD or aOR (95% CI)	P
	A/G or A/A	N	G/G	N				
Body size								
Length z-score	1.07 (1.12)	18	0.78 (0.90)	49	0.30 (−0.22, 0.81)	0.26	0.36 (−0.21, 0.94)	0.22
Weight z-score	1.18 (1.19)	18	0.75 (0.89)	49	0.45 (−0.07, 0.97)	0.09	0.42 (−0.16,1.00)	0.16
Small for gestational age								
Population	0 (0.0%)	18	0 (0.0%)	49	-	**-**	**-**	**-**
Customized	0 (0.0%)	18	1 (2.0%)	49	-	**-**	**-**	**-**
Large for gestational age								
Population	8 (44.4%)	18	12 (24.5%)	49	2.42 (0.70, 8.38)	0.16	2.68 (0.69, 9.57)	0.16
Customized	4 (22.2%)	18	6 (12.2%)	49	2.72 (0.56, 13.25)	0.21	4.75 (0.75, 30.2)	0.10
Body composition								
BMI, kg/m^2^	14.3 (2.0)	18	14.0 (1.1)	49	0.4 (−0.4, 1.3)	0.29	0.3 (−0.5, 1.1)	0.46
z−score	0.77 (1.14)	18	0.50 (0.76)	49	0.28 (−0.18, 0.75)	0.24	0.24 (−0.27, 0.75)	0.10
Skinfold thickness, mm								
Triceps	6.8 (1.7)	16	6.5 (1.6)	45	0.4 (−0.5, 1.3)	0.38	0.3 (−0.6, 1.3)	0.47
Subscapular	6.7 (1.8)	16	5.7 (1.4)	45	0.7 (−0.1, 1.6)	0.09	0.5 (−0.4, 1.4)	0.24
Arm muscle cross-sectional area, cm^2^	8.1 (6.8)	16	7.7 (7.4)	45	1.6 (−2.0, 5.2)	0.38	0.9 (−2.9, 4.6)	0.65
Whole body FM, kg	0.4 (0.2)	12	0.5 (0.2)	25	0.0 (−0.2, 0.1)	0.52	0.0 (−0.2, 0.1)	0.38
FMI, kg/m^2^	1.6 (0.7)	12	1.8 (0.8)	25	−0.2 (−0.7, 0.3)	0.37	−0.3 (−0.9, 0.2)	0.19
Whole body LM, kg	3.4 (0.7)	12	3.3 (0.3)	25	0.2 (−0.1, 0.5)	0.18	0.3 (0.0, 0.6)	0.10
LMI, kg/m^2^	12.5 (1.4)	12	12.2 (0.8)	25	0.4 (−0.3, 1.1)	0.23	0.5 (−0.3, 1.3)	0.19

Data are number (percent) or mean (standard deviation). Z-scores specific for gestation and sex. Model 1: adjusted for potential confounding by gestational age, sex, ethnicity and parity. Model 2: adjusted for potential confounding by gestational age, sex, ethnicity, parity and mediation by gestational diabetes (International Association of Diabetes in Pregnancy Study Groups diagnostic thresholds).

*FM* fat mass, *FMI* fat mass index, *LM* lean mass, *LMI* lean mass index.

**Table 3:** Body size and composition at 5 to 6 months.

Outcomes	Infant CREBRF rs373863828 genotype				Adjusted model 1 aMD (95% CI)	P	Adjusted model 2 aMD (95% CI)	P
	A/G or A/A	N	G/G	N				
Body size								
Length z-score	1.21 (1.11)	16	1.18 (1.21)	48	0.00 (−0.67, 0.68)	0.99	0.11 (−0.79, 0.58)	0.76
Weight z-score	1.19 (1.20)	16	1.03 (1.06)	48	0.08 (−0.52, 0.69)	0.79	0.10 (−0.53, 0.73)	0.75
Body composition								
BMI, kg/m^2^	18.2 (1.8)	16	17.8 (1.8)	48	0.2 (−0.8, 1.2)	0.70	0.4 (−0.6, 1.4)	0.49
z-score	0.68 (1.19)	16	0.48 (1.11)	48	0.12 (−0.51, 0.76)	0.71	0.23 (−0.42, 0.87)	0.50
Triceps skinfold thickness, mm	12.1 (1.9)	16	11.9 (3.0)	48	−0.2 (−1.8, 1.4)	0.84	−0.1 (−1.7, 1.6)	0.95
z-score	1.30 (0.90)	16	1.19 (1.36)	48	−0.07 (−0.78, 0.64)	0.84	−0.02 (−0.75, 0.71)	0.96
Subscapular skinfold thickness, mm	10.7 (2.0)	16	9.6 (2.1)	48	1.0 (−0.1, 2.1)	0.08	0.8 (−0.4, 1.9)	0.18
z-score	1.90 (0.90)	16	1.28 (1.19)	48	0.47 (−0.12, 1.06)	0.12	0.35 (−0.25, 0.94)	0.26
Arm muscle cross-sectional area, cm^2^	9.9 (2.0)	16	10.4 (2.9)	48	−0.7 (−2.1, 0.7)	0.32	−0.8 (−2.2, 0.7)	0.30
Whole body FM, kg	2.4 (0.4)	6	2.2 (0.7)	22	0.2 (−0.3, 0.6)	0.51	0.1 (−0.3, 0.6)	0.54
FMI, kg/m^2^	5.4 (0.9)	6	4.8 (1.4)	22	0.6 (−0.5, 1.6)	0.26	0.6 (−0.4, 1.6)	0.27
Whole body LM, kg	6.1 (0.7)	6	6.0 (0.7)	22	−0.2(−0.7, 0.4)	0.52	−0.2 (−0.7, 0.4)	0.52
LMI, kg/m^2^	13.5 (1.4)	6	13.2 (0.9)	22	0.3 (−0.6, 1.1)	0.57	0.3 (−0.6, 1.1)	0.51

Data are number (percent) or mean (standard deviation). Z-scores specific for gestation and sex. Model 1: adjusted for potential confounding by gestational age, sex, ethnicity and parity. Model 2: adjusted for potential confounding by gestational age, sex, ethnicity, parity and mediation by gestational diabetes (International Association of Diabetes in Pregnancy Study Groups diagnostic thresholds).

*FM* fat mass, *FMI* fat mass index, *LM* lean mass, *FMI* fat mass index.

At 12 to 18 months’ corrected age, infants with the rs373863828 A allele, compared to those without the variant allele, had lower whole-body FM [FM 1.4 (0.7) vs. 1.7 (0.7) kg, aMD −0.4, 95% CI −0.7, 0.0, P = 0.05; FMI 2.2 (1.1) vs. 2.6 (1.0) kg/m^2^ aMD −0.6, 95% CI −1.2, 0.0, P = 0.04]. Although the infants with the rs373863828 A allele, compared to those without the variant allele, had increased whole-body LM at 12 to 18 months’ corrected age of similar magnitude to the reduction in whole-body FM, this result was not statistically significant [LM 10.4 (1.8) vs. 9.7 (1.4) kg, aMD 0.4, 95% CI −0.4, 1.3, P = 0.32; LMI 16.1 (2.0) vs. 15.5 (1.7) kg/m^2^ aMD 0.4, 95% CI −0.6, 1.4 P = 0.44]. Similarly, in infants with the rs373863828 A allele, there were non-significant increases in length z-score and arm-muscle area, a measure of muscle mass. Consequently, overall body size, as assessed by BMI, was similar between groups. The reduction in FM at 12 to 18 months’ corrected age in infants with the rs373863828 A allele appeared to be related to lower abdominal fat rather than subcutaneous fat.

In secondary analysis, this association was not significant when additionally adjusted for maternal GDM (Table 4). In secondary post hoc analysis, results at 12 to 18 months were not appreciably altered by adjusting the primary model for exposure to the HUMBA Trial interventions.

**Table 4:** Body size and composition at 12 to 18 months.

Outcomes	Infant CREBRF rs373863828 genotype				Adjusted model 1 aMD (95% CI)	P	Adjusted model 2 aMD (95% CI)	P
	A/G or A/A	N	G/G	N				
Body size								
Length z-score	1.23 (1.21)	17	0.72 (1.26)	43	0.42 (−0.28, 1.13)	0.24	0.43 (−0.31, 1.16)	0.25
Weight z-score	1.57 (1.30)	17	1.25 (1.02)	43	0.10 (−0.48, 0.68)	0.74	0.17 (−0.44, 0.77)	0.59
Body composition								
BMI, kg/m^2^	18.4 (2.0)	17	18.1 (1.7)	43	−0.1 (−1.0, 0.8)	0.83	0.0 (−1.0, 0.9)	0.96
z-score	1.22 (1.26)	17	1.14 (1.01)	43	−0.16 (−0.71, 0.39)	0.57	−0.08 (−0.64, 0.49)	0.79
Triceps skinfold thickness, mm	10.1 (2.0)	17	9.7 (2.2)	42	0.0 (−1.1, 1.1)	0.95	0.1 (−1.0, 1.3)	0.82
z-score	1.06 (1.05)	17	0.90 (1.09)	42	0.00 (−0.54, 0.54)	1.00	0.07 (−0.48, 0.63)	0.79
Subscapular skinfold thickness, mm	8.6 (3.0)	17	7.6 (1.8)	42	0.8 (−0.4, 1.9)	0.21	0.7 (−0.5, 1.9)	0.25
z-score	1.23 (1.34)	17	0.77 (1.12)	42	0.30 (−0.30, 0.91)	0.33	0.32 (−0.32, 0.95)	0.33
Abdominal preperitoneal fat								
Depth, mm	3.7 (1.7)	11	4.3 (1.4)	34	−0.5 (−1.5, 0.4)	0.28	−0.4 (−1.4, 0.6)	0.42
Area, cm^2^	0.5 (0.2)	11	0.6 (0.3)	34	−0.1 (−0.2, 0.1)	0.53	0.0 (−0.2, 0.1)	0.73
Arm muscle cross-sectional area, cm^2^	22.0 (13.6)	17	19.8 (16.1)	42	0.6 (−7.4, 8.7)	0.88	0.8 (−7.6, 9.2)	0.86
Whole body FM, kg	1.4 (0.7)	17	1.7 (0.7)	40	−0.4 (−0.7, 0.0)	0.05	−0.2 (−0.6, 0.1)	0.20
FMI, kg/m^2^	2.2 (1.1)	17	2.6 (1.0)	40	−0.6 (−1.2, 0.0)	0.04	−0.4 (−1.0, 0.1)	0.15
Whole body LM, kg	10.4 (1.8)	17	9.7 (1.4)	40	0.4 (−0.4, 1.3)	0.32	0.4 (−0.4, 1.3)	0.31
LMI, kg/m^2^	16.1 (2.0)	17	15.5 (1.7)	40	0.4 (−0.6, 1.4)	0.44	0.3 (−0.7, 1.3)	0.55

Data are mean (standard deviation). Z-scores specific for gestation and sex. Model 1: adjusted for potential confounding by gestational age, sex, ethnicity and parity. Model 2: adjusted for potential confounding by gestational age, sex, ethnicity, parity and mediation by gestational diabetes (International Association of Diabetes in Pregnancy Study Groups diagnostic thresholds).

*FM* fat mass, *FMI* fat mass index, *LM* lean mass, *FMI* fat mass index.

Conditional growth in weight and length was not significantly different between infants with and without the rs373863828 A allele from birth to 5 to 6 months or 12 to 18 months, and from 5 to 6 months to 12 to 18 months (Table 5). However, conditional growth in length from 5 to 6 months was positive in infants with the rs373863828 A allele but negative in those without (Table 5).

**Table 5:** Growth in infancy.

Outcomes	Infant CREBRF rs373863828 genotype				Adjusted model 1 aMD (95% CI)	P	Adjusted model 2 aMD (95% CI)	P
	A/G or A/A	N	G/G	N				
Conditional growth SS at 5 to 6 months from birth								
Length z-score for previous length z-score	0.17 (0.81)	15	0.24 (1.01)	42	−0.15 (−0.73, 0.43)	0.62	−0.26 (−0.84, 0.33)	0.39
Weight z-score for previous weight and length z-scores	0.05 (0.90)	16	0.17 (1.00)	48	−0.15 (−0.72, 0.42)	0.60	−0.11 (−0.70, 0.47)	0.71
Conditional growth SS at 12 to 18 months from 5 to 6 months								
Length z-score for previous length z-score	0.23 (1.13)	15	−0.12 (0.90)	42	0.26 (−0.31, 0.83)	0.37	0.36 (−0.22, 0.94)	0.22
Weight z-score for previous weight and length z-scores	−0.04 (1.47)	15	−0.21 (0.78)	42	−0.13 (−0.68, 0.43)	0.65	−0.04 (−0.61, 0.53)	0.89
Conditional growth SS at 12 to 18 months from birth								
Length z-score for previous length z-score	0.26 (0.98)	17	0.03 (0.98)	43	0.16 (−0.42, 0.74)	0.58	0.20 (−0.40, 0.80)	0.51
Weight z-score for previous weight and length z-scores	0.07 (1.15)	17	−0.01 (0.93)	43	−0.11 (−0.65, 0.43)	0.69	−0.02 (−0.58, 0.54)	0.94

Data are mean (standard deviation). SS, standard score. Model 1: adjusted for potential confounding by gestational age, sex, ethnicity and parity. Model 2: adjusted for potential confounding by gestational age, sex, ethnicity, parity and mediation by gestational diabetes (International Association of Diabetes in Pregnancy Study Groups diagnostic thresholds).

## Discussion

We found that HUMBA infants of Polynesian ancestry carrying the *CREBRF* rs373863828 A allele had similar body size and composition at birth and in early infancy compared to those without the variant allele. However, by 12 to 18 months’ corrected age, infants carrying the rs373863828 A allele had lower whole-body FM by 0.4 kg or ~ 0.6 SD, with non-significant reductions in measures of abdominal but not subcutaneous fat, suggesting a possible beneficial effect on central adiposity. Despite the decrease in FM, BMI at 12 to 18 months was similar between infants with and without the rs373863828 A allele, due to a similar absolute but non-significant increase in whole-body LM.

### Physiological role of the CREBRF gene

The *CREBRF* gene is widely expressed in all tissues and encodes for a transcription factor involved in basic cell functions, such as energy metabolism and cell differentiation^9^. The minor rs373863828 A allele, possibly positively selected for in Polynesian and Micronesian populations, is known to be associated with higher BMI but a leaner body composition^11,22^, lower fasting glucose concentrations, and decreased incidence of type 2 diabetes and GDM^23^. The rs373863828 A allele is associated with increased glucose-stimulated insulin release, without an effect on insulin sensitivity, suggesting that the variant may preserve β-cell function^24^. Postprandial insulin secretion is a biphasic process, with a rapid first-phase of release followed by a reduced, sustained second phase, which continues until normoglycaemia is restored^25^. In adults, carriage of the rs373863828 A allele seems to enhance the first phase of insulin release, resulting in better glycaemic control^24^. In addition, the rs373863828 A allele may increase the capacity for insulin-independent glucose clearance in skeletal muscle, either by increasing muscle mass, the most abundant component of lean mass^26^, or by upregulating the glycolysis pathway in muscle^27^.

### Impact of the CREBRF rs373863828 A allele in neonates

The impact of the *CREBRF* rs373863828 A allele on infant growth has been less well studied. Arslanian et al*.* assessed the relationship between the *CREBRF* rs373863828 A allele and neonatal body composition (mean age 6 days) measured by dual-energy X-ray^12^. Similar to our study, they did not find any difference between neonates with and without the rs373863828 A allele in body size, whole body fat and lean mass, and skinfold thickness, even after accounting for age at assessment, sex, breastfeeding status, and maternal BMI. Neonates with the rs373863828 A allele had significantly higher bone mass, although the difference was small (+ 3 g). We additionally adjusted body composition models for maternal GDM, but this did not influence results at birth. Together, these two studies suggest that fetal carriage of the *CREBRF* rs373863828 A allele is not associated with altered size at birth, regardless of any effect of maternal rs373863828 status on glucose homeostasis in pregnancy, and that studies investigating the effect of pregnancy interventions on neonatal body size and composition in Polynesian populations are unlikely to be confounded by the *CREBRF* rs373863828 A allele.

### Impact of the CREBRF rs373863828 A allele in infancy

However, by 2 to 4 months of age, Arslanian et al*.* found that infants carrying the rs373863828 A allele had a modest increase in whole-body lean mass (+ 200 to 250 g) but no evidence of altered body fat or length^12^. We did not find any significant difference in whole-body LM at 5 to 6 months’ corrected age, although the absolute difference between groups was similar (+ 300 g), suggestive of a type II error. The study by Arslanian et al*.* may have had greater precision due to a larger sample size and use of DEXA. Consistent with these findings, we found that by 12 to 18 months, carriage of the rs373863828 A allele was associated with a non-significant increase in whole-body LM of 400 g, and results were similar after adjusting for exposure to maternal GDM.

In contrast to the data for LM, the association between the rs373863828 A allele and whole-body FM at 12 to 18 months was reduced and no longer significant after adjustment for maternal GDM. This may be explained by the fact that GDM has been associated with increased FM in infancy^28^ but in this cohort maternal carriage of the *CREBRF* rs373863828 A allele was associated with reduced incidence of GDM^14^. Future studies should evaluate further whether any potential effect of the rs373863828 A allele on FM gain in infancy is mediated, at least in part, by maternal *CREBRF* rs373863828 and GDM status, and whether this apparent leaner body composition tracks through childhood and beyond.

### Association of between the CREBRF rs373863828 A allele and linear growth

The timing of a possible effect of the *CREBRF* rs373863828 A allele on linear growth is also unclear. Carlson et al*.* found that in Samoan children and teenagers from age 5 to 18 years, the *CREBRF* rs373863828 A allele was associated with a 0.4 increase in height z-score per allele, consistent with findings in Samoan adults^13^. Although we did not find significant differences in length in infancy, conditional growth in length from birth to 12 to 18 months was positive infants who carried the rs373863828 A allele but negative in those who did not, suggesting that the variant may be associated accelerated linear growth from late infancy. It is unclear if body segment proportions are altered, although accelerated growth before puberty usually affects appendicular more than axial growth. Further research is needed to understand the biological mechanism by which *CREBRF* rs373863828 A allele may affect skeletal growth, although it is possible that this may be partly mediated by reduced exposure to GDM as GDM has previously been associated with decreased linear growth in early childhood^28^.

### Strengths and limitations

Strengths of our study include prospective data collection from early pregnancy, standardised assessment for GDM, detailed anthropometric and body composition assessment, use of conditional growth analysis and assessment of maternal genotype. The main limitation of our study is the modest sample size and the potential for type 2 error. Other limitations are that there were some missing maternal data on GDM for the secondary analysis, and that we could not explore the relationship between infant body composition and maternal BMI as all women in the HUMBA Trial were classified as having obesity.

## Conclusion

In this cohort of Māori and Pacific infants exposed antenatally to maternal obesity, carriage of the *CREBRF* minor rs373863828 A allele did not alter body size and composition at birth and early infancy but was associated with lower FM at 12 to 18 months’ corrected age, although this association may have been mediated by reduced exposure to GDM.

## Data Availability

The datasets generated during and/or analysed during the current study are available from the corresponding author on reasonable request.
